# Electrophysiological Monitoring of Brain Injury and Recovery after Cardiac Arrest

**DOI:** 10.3390/ijms161125938

**Published:** 2015-10-30

**Authors:** Ruoxian Deng, Wei Xiong, Xiaofeng Jia

**Affiliations:** 1 Department of Biomedical Engineering, The Johns Hopkins University School of Medicine, Baltimore, MD 21205, USA; rdeng3@jhu.edu; 2 Department of Neurosurgery, University of Maryland School of Medicine, Baltimore, MD 21201, USA; 3 Department of Neurology, Case Western Reserve University School of Medicine, Cleveland, OH 44106, USA; wei.xiong@UHhospitals.org; 4 Neurological Institute, University Hospitals Case Medical Center, Cleveland, OH 44106, USA; 5 Department of Anesthesiology and Critical Care Medicine, The Johns Hopkins University School of Medicine, Baltimore, MD 21205, USA; 6 Department of Orthopaedics, University of Maryland School of Medicine, Baltimore, MD 21201, USA

**Keywords:** cardiac arrest, hypothermia, prognostication, electrophysiology, EEG, evoked potentials, ischemic brain injury

## Abstract

Reliable prognostic methods for cerebral functional outcome of post cardiac-arrest (CA) patients are necessary, especially since therapeutic hypothermia (TH) as a standard treatment. Traditional neurophysiological prognostic indicators, such as clinical examination and chemical biomarkers, may result in indecisive outcome predictions and do not directly reflect neuronal activity, though they have remained the mainstay of clinical prognosis. The most recent advances in electrophysiological methods—electroencephalography (EEG) pattern, evoked potential (EP) and cellular electrophysiological measurement—were developed to complement these deficiencies, and will be examined in this review article. EEG pattern (reactivity and continuity) provides real-time and accurate information for early-stage (particularly in the first 24 h) hypoxic-ischemic (HI) brain injury patients with high sensitivity. However, the signal is easily affected by external stimuli, thus the measurements of EP should be combined with EEG background to validate the predicted neurologic functional result. Cellular electrophysiology, such as multi-unit activity (MUA) and local field potentials (LFP), has strong potential for improving prognostication and therapy by offering additional neurophysiologic information to understand the underlying mechanisms of therapeutic methods. Electrophysiology provides reliable and precise prognostication on both global and cellular levels secondary to cerebral injury in cardiac arrest patients treated with TH.

## 1. Introduction

Out-of-hospital cardiac arrest (CA) affects approximately 326,200 patients annually in the United States [1]. However, approximately 10.6% of emergency medical service-treated CA patients survive to discharge and only 8.3% have good neurological outcomes [1]. Therapeutic hypothermia (TH) has been recommended by several international guidelines as a neuroprotective method for post-CA patients after the return of spontaneous circulation (ROSC) [2,3,4,5]. However, the efficacy of existing prognostication parameters, as stated in the 2006 AAN report [6], regarding functional outcome is limited because the guidelines have not been altered to consider the increasing usage of TH.

Current neurophysiologic prognostication parameters include clinical examination, biochemical markers, neuroimaging and electrophysiological testing. Clinical examination, brainstem reflexes, and neuron-specific enolase retain their predictive robustness [7,8,9], but may lead to inconclusive results with TH intervention. With the advances of different imaging techniques, *i.e*., Computed Tomography (CT), Magnetic Resonance Imaging (MRI) and Positron Emission Tomography (PET), novel imaging markers to predict long-term neurological recovery of patients after CA have been developed [10,11,12]. Nevertheless, the low resolution, limitations in obtaining real-time information, and the lack of large-sample studies compared with other established prognostic markers make neuroimaging markers supplemental to other prognostic methods for post-CA patients. Therefore, there is a strong need to develop reliable and noninvasive tools to improve post-CA functional outcome prognostication.

We and other groups have investigated the accuracy of multimodal prognostic markers [13,14,15] with TH intervention. Among these markers, improved electrophysiological monitoring with emphasis on electroencephalogram (EEG) and evoked potentials (EPs), can help clinicians determine the degree of global neurologic injury with greater sensitivity and specificity, and can be easily implemented at the patient’s bedside. A summary of global brain monitoring markers with TH intervention from recent literatures can be found in Table 1. Cellular electrophysiology, such as local field potentials (LFP) and spikes, provides more detailed information on neuron populations and individual neurons, allowing for a better understanding of neuropathological mechanisms and improvement of therapeutic methods after cardiac arrest.

The purpose of this review is to assess the application of electrophysiology methods, identify their advantages and limitations on monitoring post-CA brain injury, and to provide a comprehensive framework for future clinical prognostication.

**Table 1:** Summary of electrophysiological research in post-cardiac arrest (CA) survivors with therapeutic hypothermia (TH) intervention.

Research Group	Background Condition of Subjects	The Timing of the Monitoring	Results
***Clinical Study***			
Rossetti *et al.*, 2010 [16]	111 consecutive comatose post-CA patients and not brain dead within 48 h TH to 33 ± 1 °C for 24 h and passively rewarming to 35 °C	Continuous electroencephalography (cEEG) and SSEPs were recorded within 72 h after CA	Unreactive EEG background was strongly associated with mortality (adjusted odds ratio for death, 15.4). The presence of at least 2 independent predictors out of 4 (incomplete brainstem reflexes, myoclonus, unreactive EEG, and absent cortical SSEP) accurately predicted poor long-term neurological outcome (Positive Predictive Value (PPV) = 1.00).
Rundgren *et al.*, 2010 [17]	111 consecutive comatose post-CA patients with a Glasgow Coma Scale score of less than 7 TH to 33 ± 1 °C for 24 h and rewarm at 0.5 °C/h	Amplitude-Integrated EEG (aEEG) monitoring was stopped if the patients showed signs of awakening, death or persistent comatose and no later than 120 h after CA	aEEG continuous pattern was highly correlated with the recovery of consciousness (29/31 patients at start of registration and 54/62 patients at normothermia). Patients with aEEG suppression-burst pattern remained comatose even dead. The aEEG status epilepticus (Negative Predictive Value (NPV) of 0.92) developing from a continuous background was found in patients who regained consciousness (2/10 patients).
Seder *et al.*, 2010 [18]	97 post-CA patients within 12 h of ROSC TH to 33 ± 1 °C for up to 24 h and rewarm to 36.5 °C within 12 h	Bispectral Index Monitoring (BIS) monitoring was recorded until rewarming was completed	The higher BIS predicted good outcome with likelihood ratio of 14.2 and an area under the curve of 0.91. Supression ratio larger than 48 predicted poor outcome with likelihood ratio of 12.7 and an area under the curve of 0.90.
Tjepkema-Cloostermans *et al.*, 2013 [19]	109 consecutive comatose post-CA patients without addition neurologic injuries TH to 33 °C for 24 h	EEG recordings were started after the patients’ arrival on the ICU and lasted up to 5 days or until discharge	At 24 h after CA, a Cerebral Recovery Index (CRI) < 0.29 predicted poor outcome (sensitivity = 0.55, specificity = 1.00, PPV = 1.00, NPV = 0.71). A CRI > 0.69 predicted good outcome (sensitivity = 0.25, specificity = 1.00, PPV = 0.55, NPV = 1.00).
Noirhomme *et al.*, 2014 [20]	46 postanoxic comatose patients The average time from CA to ROSC was 20 ± 12 min TH to 33 ± 1 °C for 24 h	Video-EEG was performed during TH for at least 20 min and repeated after rewarming	Non-reactive EEG background and discontinuous EEG background were strongly associated with poor outcome and continuous EEG was related with good outcome by automatic analysis of EEG background and reactivity.
Grippo *et al*., 2013 [21]	60 consecutive comatose post-CA patients (Glasgow Coma Scale < 9) within 60 min from collapse to ROSC TH to 33 ± 1 °C for 24 h	Somatosensory evoked potentials (SSEPs) were recorded during TH and after re-warming	None of patients with the absence of N20 regained consciousness. The patients with the absence of N20 during TH did not get the recovery of N20 after re-warming.
***Animal Study***			
Chen *et al*., 2013 [22]	20 adult rats under 5-min cardiac arrest TH to 33.5 °C for 2 h and re-warming to 37 °C over 2 h	cEEG was recorded for 6 h	Burst frequency and spectrum entropy of EEG measurement were higher in hypothermia group than normothermia group and they were highly correlated with 96-hr favorable outcome and survival.
Jia *et al*., 2008 [23]	24 adult rats under 7-min asphyxia-cardiac arrest TH to 33 ± 1 °C for 6 h and re-warming from 33 to 37 °C in 2 h Hyperthermia to 39 ± 0.5 °C and cooling to 37 °C in 2 h	cEEG was recorded hypothermia and re-warming and additon 2-h recovery period Serial 30-min recording was conducted at 24, 48 and 72 h after ROSC	Information Quantities (IQs) in normothermia group and hyperthermia were significantly lower than those in hypothermia group. The cut-off points at 30 min, 60 min, 2 h and 4 h could accurately predict good outcome, especially the cut-off point of 0.523 at 60 min with sensitivity of 81.8% and specificity of 100%.

## 2. Electrophysiological Brain Monitoring Prognostication in Post-CA patients

Although traditional clinical examination and biochemical markers are commonly used for early prognostication of neurologic outcome, they fail to provide direct measurement of the degree of underlying neuronal activation [24]. Moreover, neuroimaging is expensive and mostly described only in studies of limited sample size without comparison with other prognostic methods. Electrophysiological brain monitoring in the early phase of recovery is able to correct for these deficiencies by providing both qualitative and quantitative information, with its performance largely independent of TH effects. Usually neurologic outcome is assessed by Glasgow–Pittsburgh Cerebral Performance categories (CPC) in clinical practice with good outcome as CPC 1 (good cerebral performance) or 2 (moderate disability) and poor outcome as CPC 3–5 (3 severe disability, 4 comatose and vegetative state and 5 death) [2,4,25], and by Neurological Deficit Score (NDS) in animal studies [22,26], where higher NDS values indicate better levels of behavioral performance or neurologic function [23].

### 2.1. Global Brain Monitoring Measurement Methods

#### 2.1.1. Electroencephalography (EEG)

EEG monitoring of temporal and spatial characteristics, such as frequency and amplitude, is one of the most common methods used to provide early bedside information about clinical prognosis for post-CA comatose patients. EEG background reactivity and continuity have been recently identified as critical factors in predicting recovery or poor outcome [13,27]. New multimodal approaches emphasize the role of continuous EEG (cEEG) and its correlation with other prognostic markers, such as neuron-specific enolase (NSE) levels, as independent predictors of poor outcome [13]. Other techniques such as amplitude-integrated EEG (aEEG) [28,29] and entropy-based quantitative EEG (qEEG) [23] aim to simplify cEEG and provide information about the recovery of EEG pattern and degree of brain injury. Automated analysis has been shown to be an alternative to visual interpretation by the physician [20], but it still requires further investigation.

##### Continuous Electroencephalography (cEEG)

The importance of continuous EEG (cEEG) has been increasingly recognized in monitoring brain function and predicting early outcomes in ICU patients. It can improve prognostication at 72 h, while neurologic examination performed at that time may be inconclusive [16,30].

**Figure 1:**
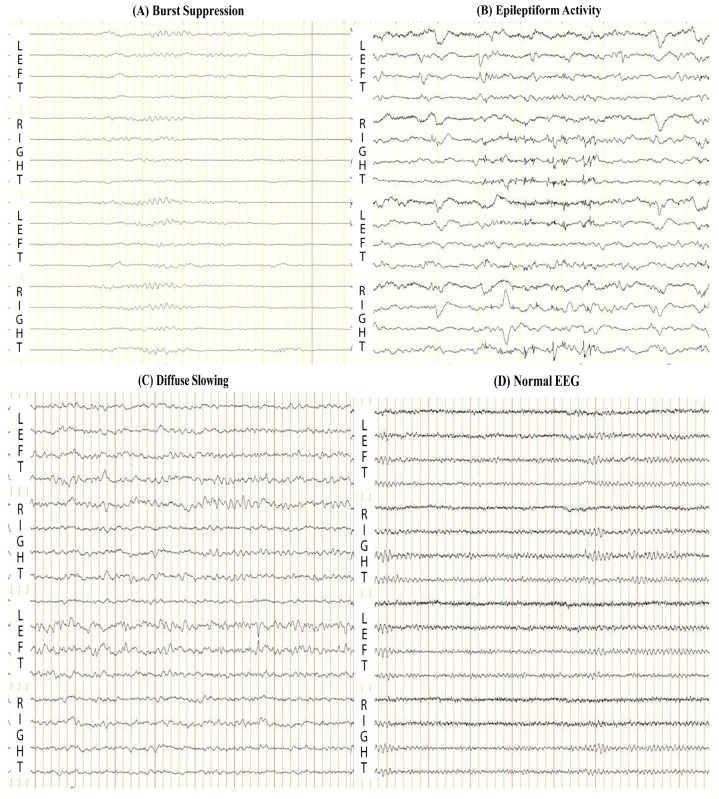
Representative abnormal electroencephalography (EEG) patterns: (**A**) Burst Suppression (the presence of bursts with amplitudes higher than 20 μV, followed by the intervals of at least 1 s with suppression of EEG activity less than 20 μV) and (**B**) Epileptiform Activity (including seizures and generalized periodic discharges) are associated with poor outcome; (**C**) Continuous Diffuse Slowing (EEG activity with a dominant frequency less than 8 Hz) and (**D**) Normal EEG at 12-h after resuscitation are associated with good outcome.

cEEG is able to predict both good and bad outcomes based on different patterns: isoelectric (defined as no visible EEG activity), low-voltage (defined as EEG activity less than 20 μV), burst suppression (Figure 1A), epileptiform activity (including seizures and generalized periodic discharges) (Figure 1B), diffuse slowing (Figure 1C), or normal (Figure 1D) [27,31]. Isoelectric and low-voltage are associated with poor outcome [27], whereas patients with good functional outcome showed normal or diffuse slowing on 12-h post-resuscitation EEG, which was never found in patients with poor outcome [27,31]. Burst suppression and epileptiform activity is associated with poor outcome, but they are not reliably predictive [6,27,32,33].

cEEG background reactivity was defined as present if brain electrical activity of at least 10 μV and EEG background indicated the alternation in amplitude or frequency on repetitive auditory, visual and nociceptive stimulus [16,34]. The presence of EEG reactivity was shown as a predictor of recovery of consciousness (all survivors had background reactivity) and favorable outcome (74% of survivors), whereas the absence of reactivity was highly associated with mortality (Positive Predictive Value (PPV) = 1, False-positive Rate = 0) [34].

Cloostermans *et al.* [27] found that cEEG was the most predictive of prognosis for poor outcome between the first 12–24 h after CA and that it offered significantly better sensitivity than other electrophysiological parameters such as somatosensory evoked potentials (SSEPs) (40% for cEEG compared to 24% for SSEPs). A prospective cohort study with 134 post-CA patients testing a multimodal approach for prognostication found that hypothermic EEG background reactivity was the best predictor for poor outcome with sensitivity of 74% and had the highest area under receiving operator characteristics curve of 0.81 [13]. The results showed that cEEG together with clinical examination and NSE levels had the highest prognostic accuracy for mortality and poor outcome in this study [13]. Another study with comatose patients showed correlation between unreactive EEG backgrounds and elevated NSE levels, suggesting that early EEG abnormalities in the first 24–48 h are pathophysiologic signs resulting from post-anoxic neuronal injury [35]. However, early presence of background reactivity does not guarantee recovery, as some patients with reactive but discontinuous backgrounds died after return to normothermia [36]. Similarly, status epilepticus, which was previously regarded as a certain predictor of poor outcome, is now shown to depend upon the type of background reactivity of EEG as being flat, burst-suppressed, or continuous [6,32,33]. In a retrospective study of 83 post-CA patients undergoing TH monitored by cEEG, patients with good outcomes had cEEG patterns categorized as diffuse slowing and epileptiform waves, however, these classifications still require subjective interpretation of the cEEG [31]. Isolated cEEG use could be useful in the first 24 h [33], but it is preferable that it be used as part of a multimodal approach, whereby other neurophysiological factors are included, such as SSEP and neurologic examination.

Large animals, like piglets, have been used as an ideal alternative model for CA research [37,38,39] due to their high tolerance of invasive experimental procedures and similar physiology and cerebral anatomy to humans [40]. Agnew *et al.* [38], has shown that cEEG recovery was similar in animals with the use of sedation and paralysis in normothermia and hypothermia. The prognostic value of cEEG could be better elucidated through future swine studies on CA.

##### Quantitative Electroencephalography (qEEG)

EEG monitoring methods, such as EEG background and reactivity, hold important prognostic value about the neurological outcome of patients treated with TH [35], but prognostication is often confounded by subjective interpretation by neurologists who review the EEGs. For these reasons, quantitative EEG (qEEG) techniques were introduced, *i.e.*, aEEG, Bispectral Index (BIS) monitoring, Cerebral Recovery Index (CRI), and entropy-based qEEG, showing objective and accurate prognostication and bringing new insights into EEG monitoring in clinical practice.

##### qEEG: Amplitude-Integrated EEG (aEEG)

The amplitude-integrated EEG (aEEG) method is increasingly used for continuous brain function monitoring of post-CA patients, as its interpretation requires less time and expertise [41]. It is a simplified method that shows the peak-to-peak amplitude values of rectified EEG on a time-compressed semi-logarithmic scale [41,42]. This method has been evaluated as a prognostic tool for predicting outcome in post-CA adult patients. Rundgren’s group investigated 34 TH-treated post-CA patients: 20 patients with a continuous aEEG after rewarming returned to consciousness, while others with abnormal aEEG patterns did not return to consciousness and died in hospital [28]. The same group in 2011 further showed the early predictive ability of aEEG for good and poor outcome and found that a continuous background was associated with good outcome in 111 post-CA survivors treated with TH [17]. In a prospective study of 55 TH treated post-CA patients, an initial continuous normal voltage (CNV) tracing of the aEEG (upper margin voltage > 10 μV and lower margin voltage > 5 μV) immediately after ROSC was a good predictor of good outcome (sensitivity: 57.1%, specificity: 96.3%), while a lack of CNV in the first 72 h after ROSC was a good predictor of poor outcome (sensitivity: 77.8%, specificity: 100%) [29]. Another piglet study showed the suppression of upper and lower margins of aEEG increased with the severity of brain injury [43]. Together these results demonstrate that aEEG is able to provide practical information on the degree of cerebral dysfunction, which can be regarded as a simpler and more reliable applied method in an ICU setting compared to standard cEEG monitoring.

##### qEEG: Bispectral Index (BIS) Monitoring

Bispectral Index (BIS) monitoring is a technique that summarizes raw EEG data and is commonly used to evaluate consciousness under anesthesia. It has been suggested that BIS is a poor index for evaluation during CPR and for the prediction of ROSC [44,45], and the technique has inconclusive data regarding its prognostic ability after CA, and thus, is not widely used. In a prospective study of 62 post-arrest patients that underwent BIS monitoring following TH, mean BIS values were higher in good-outcome patients compared to poor-outcome patients at 24 h post-resuscitation [46]. The study suggests that BIS values at 24 h post-resuscitation are correlated with neurologic outcome in patients receiving TH after CA. In another study of 97 patients with BIS monitoring following CA and TH, the BIS after the first administration of neuromuscular blockade was higher in patients with good outcomes compared to those with poor outcomes [18]. A prospective cohort study of 45 comatose patients that had received TH after CA, found that a BIS of 0 was a good predictor of poor neurologic outcome with PPV of 100% and Negative Predictive Value (NPV) of 55% for good outcome when BIS was not 0, however, there was no correlation between a BIS higher than 0 and good outcome [47]. This technique may be beneficial due to its simplicity; however, the conflicting results in terms of prognostic ability suggest that further research is necessary.

##### qEEG: Cerebral Recovery Index (CRI)

The Cerebral Recovery Index (CRI) was developed to assist clinicians with the prognostication of patients treated with TH after CA and is a single numeric value that represents five qEEG parameters. In an initial single-center study of 109 patients treated with TH post-CA and cEEG monitoring, at 24 h post-CA, CRI < 0.29 was associated with poor outcome (PPV = 1, NPV = 0.71) while CRI > 0.69 was associated with good outcome (PPV = 0.55, NPV = 1) [19]. This index is beneficial as it uses the clinically important information within EEG and can predict both good and bad outcome. However, given the lack of clinical data using this index, it should be used only as a complementary criterion in prognostication.

##### qEEG: Entropy-Based Quantitative Electroencephalography

A new automatic method based on quantitative characteristics, such as Burst Suppression Ratio (BSR) and approximate entropy, has been developed to measure background activity in the clinical setting [20]. The results between the visual and automated analysis correlate well, but the automatic approach was influenced by epileptiform activity and muscle artifacts among others, which required the expertise of physicians to detect the suppressed state. The same study also confirmed that BSR and approximate entropy differ significantly for good and poor outcome, with reactivity being the stronger factor compared to background discontinuity. A comparative study with 30 post-CA patients who were under TH of 33 °C for 24 h revealed that elevated response entropy and state entropy during the first 24 h were associated with good outcome, while decreased wavelet sub-band entropy (WSE) and increased BSR in the first 24–48 h were strong indicators for poor outcome [48]. The same study also confirmed that status epilepticus, represented by decreased WSE levels, was a certain predictor of mortality.

Another animal study showed that increased burst frequency, shorter isoelectric period, and preserved spectrum entropy after the restoration of continuous background activity were associated with good outcome and survival at 96 h [22]. On the other hand, Jia *et al.* [49], found that burst frequency was higher in the post-CA animals treated with TH and hyperthermia compared to normothermia. The burst frequency was strongly correlated with 72-h neurologic function at normothermia but not in animals treated with TH or hyperthermia. However, burst counting was time-consuming and underestimated some essential information, such as the duration of burst or suppression duration. For these reasons, the same group developed quantitative measures of BSR using Tsallis entropy (TsEn) and revealed that the TsEn area correlated well with functional outcome [50]. However, more research is required including on the effects of temperature management before these results can be translated into clinical practice.

**Figure 2:**
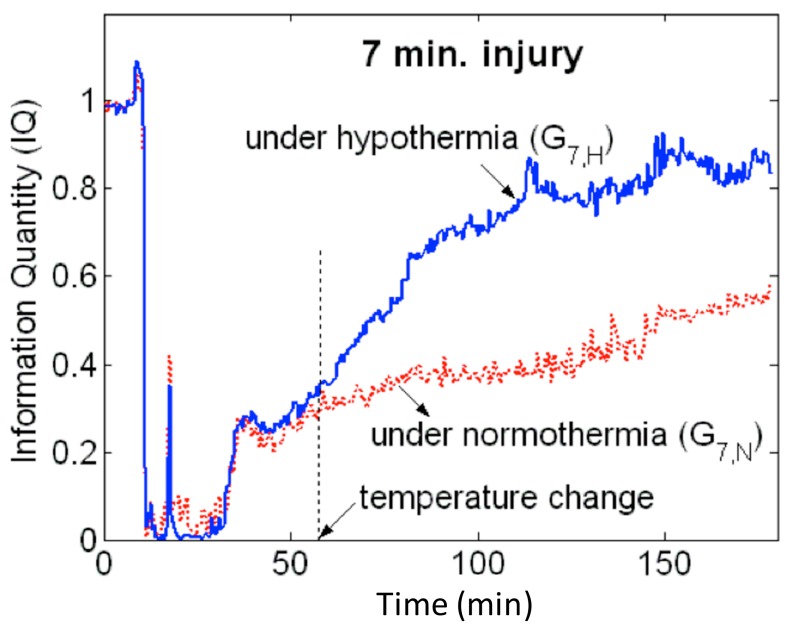
Information Quantity (IQ) temporal evolution following a 7-min CA model in rats. The blue curve represents the IQ profile when TH was applied at the point marked as “temperature change.” The red curve represents the IQ profile when normothermia was maintained. At the onset of injury, there is a rapid drop of IQ values, but after the temperature change, the blue curve shows an increasing trend of higher IQ values compared to the red curve, displaying the protective effect of TH compared to normothermia.

Another quantitative metric that has shown promising results in animals regarding differentiating good and poor outcome and the effect of temperature on recovery of cortical electrical activity is information quantity (IQ) [23,51]. Based on information theory, IQ calculates Shannon entropy based on a distribution of all wavelet coefficients and subsequent removal of the predictable component (information redundancy) using discrete wavelet transformation (DWT). IQ was able to accurately predict the impact of temperature on functional outcomes and mortality soon after resuscitation (Figure 2). However, IQ can only distinguish changes in gross wide-band EEG. Sub-band IQ (SIQ) was then introduced to calculate the IQ of each frequency sub-band, relating behavioral state to recovery possibility [52]. The recovery of gamma-band SIQ was an early predictor of functional outcomes at 72 h after resuscitation in an animal model of CA. Induced TH enhanced fast recovery of gamma-band SIQ and improved functional outcomes [53]. Further verification of this method, nevertheless, needs to be studied on humans in the clinical setting.

#### 2.1.2. Evoked Potentials (EPs)

Although EEG monitoring is generally easy to conduct and interpret, it can be affected by external factors such as medications and sedation in the first three days after ROSC and does not provide detailed information about the degree of injury to specific central nervous system pathways in the brain. Evoked potentials (EPs) provide information about the degree of functional damage of the different neurologic pathways: somatosensory, motor, auditory, and visual. EPs can be used to predict prognosis and is more robust to sedation, although is still affected by muscle artifacts and relaxation [54].

##### Somatosensory Evoked Potentials (SSEPs) SSEPs: Waveform-based SSEPs

**Figure 3:**
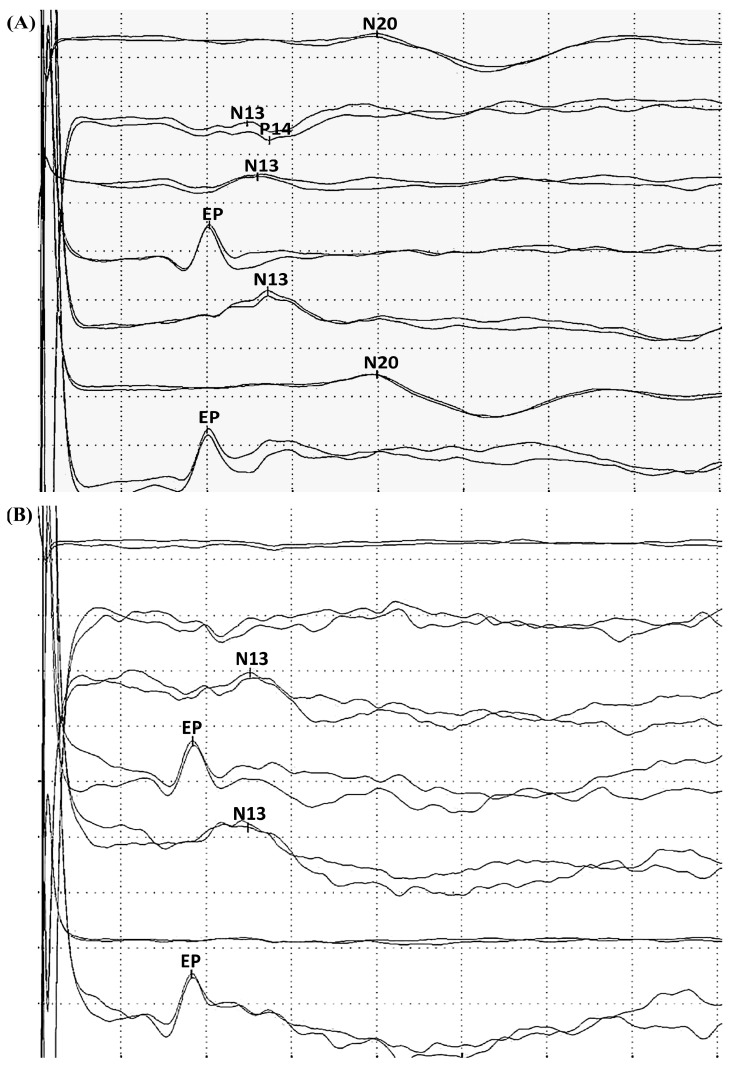
Example of (**A**) normal somatosensory evoked potentials (SSEPs) cortical N20 response to median nerve stimulation, demonstrating the integrity of the somatosensory pathway; and (**B**) SSEPs with absent N20, which has been regarded as a reliable predictor for poor outcome in patients after CA.

SSEP (Figure 3A) can be used to assess the integrity of the somatosensory pathway, the restoration of normal thalamocortical coupling, and the onset of arousal [55]. Experiments have demonstrated that a significant and measurable difference existed in SSEP signals based on neurologic injury and that SSEP evolved in a predictable manner, which correlated with outcome in rats after CA [56]. These experiments lay the groundwork for establishing the relationship between SSEPs and post-CA neurological injuries and functional outcomes.

According to the AAN guidelines, absence of N20 components from SSEPs bilaterally (Figure 3B) up to three days after resuscitation has been found to be the most reliable electrophysiological biomarker for mortality or poor outcome [6]. Specifically, the absence of the N20 potential after stimulation of the median nerve under certain conditions is indicative that the patient will never regain consciousness [57]. New theories promote SSEP in clinical settings to monitor patients resuscitated from CA not only after but also during TH intervention [9]. It has been shown that the absence of cortical SSEP components (N20) after stimulation of the median nerve after resuscitation is a reliable electrophysiological indicator of an unfavorable prognosis even after TH [58]. Another recent study with 60 patients indicated that SSEPs retained high prediction value for poor neurological outcome despite TH intervention. In patients with preserved SSEP, no significant differences were found between N20 mean amplitude during TH (6–24 h after CA) and after re-warming. In contrast, the 24 patients who showed bilaterally absent N20 did not recover cortical responses even after re-warming. All patients with absent cortical SSEPs during TH did not regain consciousness [21]. However, the use of SSEP during TH for prognostication has not yet been validated in a large hypothermic patient population.

##### SSEPs: Quantitative SSEPs (qSSEPs)

Although the prognostic value of absent N20 for poor outcome is well validated, the clinical application is limited to studying only the presence or absence of the N20 waveform and the value of the N20 for predicting good outcomes remains inconclusive. An animal study showed that quantified amplitudes and latencies of N10 (the rat equivalent to the human N20) were able to objectively track brain injury after CA and were predictive of poor and good outcomes, with higher amplitudes of N10 in rats correlating with shorter CA times and better function [56]. Madhok *et al*. [24], applied this method on uninjured rats under TH revealing that TH significantly increased amplitude and latency of N10. This study indicated the potential of using SSEP amplitude for predicting outcomes after CA under TH. However, SSEPs are only a few microvolts in amplitude and are often contaminated by biological and electrical noise, even after averaging. In order to solve the above problems, the area enclosed by the SSEP in the phase space, a space of all possible configurations of magnitudes and slopes in a signal, was investigated and introduced as a quantitative descriptor for SSEP in an animal study [55]. Advanced neural monitoring systems with novel quantitative tools such as PSC (the phase space curve) and/or PSA (the phase space area) can provide real-time markers that do not require expert interpretation and offer clinicians the option to select windows of interest (e.g., 1–4 h post-asphyxia) to study different components of SSEPs. They can also simultaneously provide a better means to track injury, recovery, and the effect of neuroprotective interventions in real-time and a neurophysiologic means of evaluating the integrity of deep-brain regions [55]. An animal study from the same group showed that PSA can accurately detect the benefits of TH and strongly correlated with good outcome [59]. However, before PSA can be adopted in routine clinical settings, further validation and studies are necessary. Other quantitative SSEP (qSSEP) techniques including quantitative sensory testing, which determines the threshold and sensation for sensory stimuli, has been applied to quantitatively detect and diagnose the functional disorders in peripheral nervous system but has not been employed on post-CA patients [60].

Another quantitative technique, second order blind identification (SOBI), was introduced to extract characteristic peak information from one single trial from SSEPs to solve noise problems in another animal study. This method can efficiently detect the peak of N10 in rats preserving variability between successive peaks and enhancing temporal resolution [61]. Since SSEPs are composed of short-latency (SL) and long-latency (LL) responses, which reflect the functional recoveries of different cortical regions, independent component analysis (ICA) was used to separate the SL and LL SSEPs, reducing their interference and improving the accuracy in detecting peaks after CA. The change of SL and LL responses demonstrated the functional dynamics of their sources of origin [62]. These techniques might have a role as a complementray tools to qSSEP, which can improve the accuracy of predictive values for post-CA outcomes. qSSEP, similar to quantitative EEG, may provide reliable and objective prognostication for both good and bad outcome in the early recovery stage of CA, but its true utility remains unclear due to the limited number of studies and the lack of clinical trials.

##### Other Diagnostic Evoked Potentials Markers

Other types of evoked potentials have not been thoroughly investigated although there has been some research on auditory evoked potentials (AEPs), motor evoked potentials (MEPs), event-related potentials (ERP) and their predictive values.

AEPs provide information about the degree of preservation of the higher auditory cortical pathways and might help determine the likelihood of both poor outcome and chance of awakening [63]. Depending on its origin, AEPs can be classified as brainstem AEPs, middle-latency AEPs (MLAEPs), and event-related AEPs (ERPs), the last being able to differentiate between different acoustic stimuli. It has been suggested that the presence of mismatch negative (MMN) waves in ERPs is predictive of imminent awakening of comatose patients, although it does not guarantee full functional recovery [63]. A recent study, however, contended that the discriminatory performance in survivors and non-survivors is not significantly different in both TH and normothermia conditions, while the change in the area under the curve in the transition from TH to normothermia is increased in survivors compared to non-survivors [64]. The study suggested that MMN presence could be observed in people with similar states of unconsciousness and could not be predictive for the final outcome, but that if the discriminatory performance changes positively in the early phase of coma, awakening will occur with 100% predictive value. The P300 is the most common ERP detected when a subject responds to a stimulus rapidly and correctly [65]. The presence of P300 has been linked with the recovery of consciousness in post-CA patients [66] and the amplitude of P300 was significantly higher in hypothermia-treated patients than normothermia-treated patients [67], but the field is not mature enough for clinical prognostication of patient outcomes. Furthermore, middle-latency AEPs (30 ms) have also been shown to exhibit changes to novel auditory stimuli [68], which is particularly important for monitoring anesthesia. A recent study from 2013 hypothesized that MLAEPs would be able to detect ROSC, survival, and neurologic outcome [69]. The research group used an MLAEP monitor to determine the MLAEP index (MLAEPi) of 61 comatose patients. The MLAEPi reflected the morphology of MLAEP, and the presence of the P50 component suggested good neurologic outcome after resuscitation [69,70]. A cut-off point of 35 was established to be the threshold above which favorable outcome is detected with 100% sensitivity while cut-off points of 24 and 33 were able to predict ROSC and post-resuscitation survival, respectively [69]. AEPs have significant potential for predicting good outcome, which is often elusive using the standard multimodal approach [13], but studies with larger group of patients are needed to determine the reliability of this method.

Presence of MEPs has long been considered to be an accurate measure of the degree of functionality of the descending cortical pathways and determine the status of the motor cortex. However, due to their high degree of sensitivity to anesthesia levels of isoflurane and ketamine and to stimulation intensity, MEPs are difficult to monitor. Previous studies have focused on using a moving average method to characterize the changes in MEP amplitudes, but this method assumes that all signals are identical, which might not be the case [71]. In order to circumnavigate this issue, a statistical method has been employed to characterize MEPs by estimating the number of motor units and the potential amplitude of a single motor unit instead of using a moving average [72]. The recordings showed that a decrease of anesthesia intensity resulted in an increase of MEP signal amplitude. This technique may have potential uses in post-CA patients, though currently there lacks any study validating this modality on such patients.

#### 2.1.3. Global Electrophysiological Prognostic Test in Neonates with Hypoxia-Ischemic Encephalopathy

Electrophysiology has been regarded as an outcome prognostic tool and real-time brain injury monitor not only in post-CA adult patients but also in hypoxic-ischemic neonates. A systematic review of 259 related studies conducted by Laerhoven *et al.* [73], highlighted aEEG background (sensitivity 0.93, specificity 0.90), EEG pattern (sensitivity 0.92, specificity 0.83) and visual evoked potential (sensitivity 0.90, specificity 0.92) as the most promising prognostic markers in term neonates with HI encephalopathy. However, SSEPs in the 1st week of life predicted neurological outcomes with a sensitivity of 0.93 and specificity of 0.78 [73]. Among these markers, aEEG [74] and SSEPs [75] have been validated as reliable predictive markers in neonates treated with TH for hypoxic-ischemic injury. In terms of aEEG, Shah *et al.* [74] showed that the presence of seizure patterns and abnormal aEEG background in first 24 h (burst suppression, continuous low voltage or isoelectric) was highly associated with brain injury (Pearson χ^2^ coefficient: 12.86 and 15.31, respectively, *p*-value < 0.001). Garfinkle *et al.* [75], reported that SSEP still retains its high predictive value with TH intervention and that the absence of N20 is able to predict neurodevelopment impairment with PPV of 0.36 and NPV of 0.93.

### 2.2. Cellular Brain Monitoring Measurement Methods

#### 2.2.1. Local Field Potentials (LFPs)

Electrophysiological brain monitoring has been expanded to include measurement of transmembrane currents and more localized events such as local field potentials (LFPs) and spikes, which can provide further information in addition to traditional EEG recordings on cellular electrophysiology and the underlying neuronal pathways. LFPs record the electric potential in the extracellular space around neurons, originate from the depolarization of groups of neurons, and are obtained by low-pass filtering (<300 Hz), while spikes are the high-frequency component of the signal representing individual action potentials. The recordings are obtained using extracellular microelectrodes to measure synaptic current events over a very small-localized brain region. LFPs can precede the occurrence of action potentials and could potentially be helpful for evaluation of the state of connectivity of neural pathways, while spike temporal characteristics could signal the presence of EEG abnormalities.

In order to study the dynamics of the HI injured brain, indexes have been developed to measure anesthetic depth to quantify and understand the electroencephalogram signal and the ongoing neural processes. LFPs present advantages over the EEG diagram, as EEG is often corrupted by high frequency artifacts and electromyographic activity [76]. In contrast to the EEG, in which typically only components below 70 Hz are studied, LFPs cover a wide range of neural signals with frequencies between 1 to 300 Hz. The multitude of excitatory and inhibitory neural processes and the band-limited structure of the signals, such as gamma, alpha and beta, provide a more holistic picture of information processing in the brain during injury and recovery. For instance, high-frequency gamma-band components are preserved in LFPs and allow a better measurement of anesthetic depth compared to only the EEG. Furthermore, LFPs can be used to assess the functionality of the cortical pathways and analyze the phase-relations between the thalamus and the cortex [77,78]. A study that analyzed thalamocortical interactions discovered that at steady state there is a high degree of coherence between the thalamus and the cortex [76]. Silva *et al*. [76] also recorded LFPs and revealed that the permutation entropy (PE) corrected for the burst suppression (BS) component is the most indicative component for quantifying anesthetic depth. Another study used LFPs to measure the vulnerability of the somatosensory responses during injury and recovery as well as during TH and normothermia [79]. The results suggested that the cortical function is more susceptible to HI injury than the subcortical regions.

#### 2.2.2. Single and Multi-Unit Activity

Action potentials can be recorded with high spatial resolution from microelectrode arrays that are implanted on the cortex, and can be separated offline into single and multi-unit activity (SUA, MUA) [80]. The raw data are high-pass filtered followed by the application of spike detection to identify single spikes. Spike sorting is then applied to the data to discriminate SUA from MUA. The activity from single neurons will be categorized by templates such that SUA is extracted from spike sorting whereas MUA will not produce unique spike clusters after the sorting process and will instead contain spikes from multiple neurons [80,81]. MUA provides more information than single-unit activity (SUA) as it accounts for smaller amplitude spike activity of the neurons around the microelectrode [82]. Since MUA is generated by the spiking of several neurons, this technique allows the study of interactions between neurons. Using a scheme of filtering and multi-resolution entropy (MRE) analysis allows for spike detection and extraction of information based on entropy levels. It has been shown that employing entropy-based resolution on the MUA signal from both simulation and real-time recordings can indeed be useful to describe cortical neuron dynamics [83]. Another study attempted to investigate the relationship between the burst suppression pattern and neural activity by using MUA, which may further elucidate the cellular mechanism in post-CA brain recovery [84]. The results indicated that the cortical firing rate is extraordinarily high and that the number of firing neurons is highest during EEG bursts. The thalamic activity recovers earlier than the cortical activity, and the correlation between these two activities increases during burst suppression after CA. LFP and MUA both underlay critical cortical processes and are interdependent, although they may differ in temporal coherence.

## 3. Conclusions

TH has been shown to be an effective treatment for out-of-hospital CA patients to enhance their survival and neurological function. The currently proposed multimodal approach of combining clinical findings, NSE levels, and electrophysiology holds promise to facilitate early prognostication of poor outcome in post-CA patients. However, the accuracy of these prognostic methods has been challenged when used in conjunction with TH, and must be further tested by studies with larger patient groups. Based on the available information, we recommend that electrophysiological brain monitoring should be conducted in the early stages after HI brain injury. Among several electrophysiological markers (Table 2), EEG background reactivity and N20 are good prognostic indicators of neurologic outcome. Additionally, qEEG and qSSEP are able to provide accurate and objective information on the degree of HI injury, allowing for early prediction of neurologic outcomes while potentially eliminating subjective errors by clinicians. However, comparison of the clinical advantages across different electrophysiological modalities should be taken with caution because studies were performed under different conditions, such as rewarming rate, patient inclusion and exclusion criteria, or signal recording duration, even though they generally used standard protocols of TH treatment (Table 1). Cellular electrophysiology, such as MUA and LFP, have strong potential for improving prognostication and therapy by assessing the thalamocortical network integrity and by offering additional cellular information to understand the underlying mechanism of therapeutic methods. These techniques may bring new insights into prognostication after CA, but they still require further investigation before being translated into clinical practice.

**Table 2:** Electrophysiological markers with pros and cons for use in prognostication.

Category	Markers	Pro	Con
cEEG	Isoelectric, low-voltage; Burst and suppression, Epileptiform pattern (not always reliable); Absence of EEG reactivity.	Directly provide measurement of neuronal activities; Low financial cost, bedside and non-invasive monitoring; EEG reactivity and continuity have been validated as critical factors in predicting recovery or poor outcome.	Confounded by subjective interpretation by neurologists; Affected by external factor, *i.e.*, medications and sedation; Not able to provide detailed information about the degree of injury.
Quantitative EEG (qEEG)	Burst suppression, continuous low voltage or flat trace EEG background and seizure pattern; Absence of aEEG continuous normal voltage (CNV) pattern; The lower values obtained from other qEEG measurement are associated with poor outcome.	Simpler, objective and accurate prognostication; Do not need neurologists’ interpretation.	Most qEEG markers (*i.e.*, CRI and Entropy-base qEEG) lacks clinical data, or larger patient groups (*i.e.*, aEEG) which needs further clinical study and can only regard as a complementary criterion in prognostication; Conflicting results (*i.e.*, BIS) of prognostic ability.
SSEPs	Bilateral absence of N20 potentials.	Provide direct information about the degree of functional damage of somatosensory pathway; Most accurate marker of poor outcome prognosis; Most robust to sedation.	Only limited to studying the presence or absence of N20; The prognostic value of good outcome is inconclusive.
Quantitative SSEPs (qSSEPs)	The lower qSSEP values are associated with poor outcome .	Do not require experts’ interpretation; Allow researchers to choose the time period of interest; Objectively provide not only prediction of bad outcome but good outcome.	qSSEP techniques lack clinical validation.
Other EPs	The absence of mismatch negative (MMN) waves in evoked-related auditory evoked potentials (ERPs); The disorder of auditory discrimination capabilities.	Provide direct information about the degree of functional damage of different neurologic pathway.	Their prognostic abilities have not been validated on TH-treated patients.
